# Multilocus Sequence Analysis and Detection of Copper Ion Resistance of *Xanthomonas phaseoli* pv. *manihotis* Causing Bacterial Blight in Cassava

**DOI:** 10.3390/cimb45070342

**Published:** 2023-06-28

**Authors:** Tao Shi, Chaoping Li, Guofen Wang, Guixiu Huang

**Affiliations:** Key Laboratory of Integrated Pest Management on Tropical Crops, Ministry of Agriculture and Rural Affairs, Environment and Plant Protection Institute, Chinese Academy of Tropical Agricultural Sciences, Haikou 571101, China; shitaofly2008@163.com (T.S.); lichaoping2008@163.com (C.L.); guofenwang7810@163.com (G.W.)

**Keywords:** cassava bacterial blight, *copLAB*, copper resistance, genetic diversity, *xmeRSA*

## Abstract

Cassava (*Manihot esculenta* Crantz) is an important tropical tuber crop around the world. Cassava bacterial blight, caused by *Xanthomonas phaseoli* pv. *manihotis*, is a key disease that influences cassava production worldwide. Between 2008 and 2020, 50 *X. phaseoli* pv. *manihotis* strains were isolated from diseased plant samples or acquired from China, Uganda, Cambodia, Colombia, Malaysia, and Micronesia. Using multilocus sequence analysis, the genetic diversity of *X. phaseoli* pv. *manihotis* strains was evaluated. A neighbor-joining phylogenetic dendrogram was constructed based on partial sequences of five housekeeping genes (*atpD*-*dnaK*-*gyrB*-*efp*-*rpoD*). The strains clustered into three groups whose clusters were consistent with *atpD* and *RpoD* gene sequences. Group I contained 46 strains from China, Uganda, Cambodia, and Micronesia, and the other two groups were comprised of strains from Colombia and Malaysia, respectively. The resistance of all these strains to copper ion (Cu^2+^) was determined, the minimal inhibitory concentration was between 1.3 and 1.7 mM, and there was no significant difference between strains from different geographic region. During genome annotation of the *X. phaseoli* pv. *manihotis* strain CHN01, homologous gene clusters of *copLAB* and *xmeRSA* were identified. The predicted amino acid sequences of two gene clusters were highly homologous with the copper-resistant protein from *Xanthomonas* strains. *CopLAB* and *xmeRSA* were amplified from all these strains, suggesting that the regulation of copper resistance is associated with two distinct metabolic pathways. *CopLAB* and *xmeRSA* were highly conserved among strains from different geographic regions, possibly associated with other conserved function.

## 1. Introduction

Cassava (*Manihot esculenta* Crantz) is an important tropical tuber crop widely cultivated in Africa, Latin America, and Asia. It is the third-largest source of calories for more than one billion people in the tropics. In many developing countries, cassava is a common economic crop. In 2021, the total harvest area of cassava was 296.52 thousand km^2^, and the yield was 314.81 billion kg [1].

Cassava was first introduced in Guangdong province of China around the 1820s. Now, cassava is widely cultivated in southern China, and experimental introduction was successfully finished in Henan, Shandong, and other northern provinces. Guangxi province currently accounts for the largest cultivation area of cassava in China, followed by Guangdong, Hainan, and Yunnan provinces. In 2021, the cultivation area was 3027.80 km^2^, and the dry chip yield was 4.95 billion kg [1]. In China, most of the cassava yield is used for the production of starch, ethanol, and many other products, and it plays a significant role in local industry. However, China is not self-sufficient in cassava production and became the largest importer of cassava from 2005.

Cassava bacterial blight (CBB) caused by *Xanthomonas phaseoli* pv. *Manihotis* (previously also known as *Xanthomonas campestris* pv. *manihotis* or *Xanthomonas axonopodis* pv. *manihotis*) is a disease that impacts cassava production worldwide. CBB was first reported in 1912 by Bondar in Brazil and has now spread to 48 main cassava-planting countries located in tropical and part subtropical regions [2]. It is identified by the presence of angular, water-soaked leaf lesions, blight, partial or total wilting of the branches, dieback, and necrosis of some vascular strands of the stems. Diseased plants show lowered growth potential or even death, usually leading to 12% to 100% yield losses [3,4]. In 1980, CBB was first identified in China in a cassava nursery located in Danzhou, Hainan and then detected in Guangdong and Guangxi provinces [5]. Recently, a comprehensive disease survey showed that CBB is popular in the major cassava-planting areas in China and has become the most destructive disease, the main cassava cultivars being susceptible [6].

The most appropriate and realistic approach for controlling crop disease is through host resistance, so genetic variation is very important for understanding the breakdown of resistance to the pathogen. Multilocus sequence analysis (MLSA), a joint analysis of multiple conserved genetic loci, is a commonly used technique for species definition in bacteriology, including reevaluation of species, pathogen identification, classification, and phylogenetic analysis [7,8,9]. The housekeeping genes *atpD* (F0F1-ATP synthase β subunit), *dnaK* (molecular chaperone), *glnA* (glutamine synthase), *gyrB* (DNA gyrase β subunit), *efp* (elongation factor P), *rpoD* (RNA polymerase sigma-70), *abc* (ATP-binding cassette), and *fyuA* (TonB-dependent receptor) have been frequently used in MLSA-based phylogenetic analyses. Ntambo et al. (2019) amplified and sequenced five housekeeping genes—*gyrB*, *glnA*, *rpoD*, *atpD*, and *abc*—in 14 *Xanthomonas albilineans* samples collected in China. MLSA-based phylogenetic analysis showed these samples and 13 other strains (not from China) were distributed in two distinct clades [10]. The population structure of 50 *Xanthomonas campestris* pv. *Campestris* (*Xcc*) strains isolated from infected cabbage was examined by MLSA of four genes—*atpD*, *fyuA*, *gyrB*, and *rpoD*—to generate 12 allelic profiles. Similar diversity of these 50 *Xcc* strains was observed using repetitive-DNA-sequence-based PCR (rep-PCR) simultaneously, and 31 strains showed the same allelic profiles [11].

Restrepo et al. (2000) assessed the genetic diversity of 238 *X. phaseoli* pv. *manihotis* strains from Colombia. Based on restriction fragment length polymorphism, amplified fragment length polymorphism and rep-PCR data, these strains were clustered into 17, 18 and 4 groups, respectively [12]. The draft genomes of 65 *X. phaseoli* pv. *manihotis* strains from 11 countries were obtained using Illumina deep-sequencing technology, and the identified single-nucleotide polymorphisms (SNPs) were concatenated, aligned, and used to build a phylogenetic tree based on a neighbor-joining analysis, which showed that most strains clustered by geographic origin [13]. The population structure of *X. phaseoli* pv. *manihotis* from other countries has also been studied [14,15,16]. However, phylogenetic analyses on *X. phaseoli* pv. *manihotis* strains of China remain limited.

Copper-based bactericides are effective against plant bacterial diseases and are extensively used in the field. In China, farmers are recommended to use copper-based bactericides for CBB. The inhibitory effect of two kinds of frequently used bactericides, 20% thiadiazole copper SC and 77% copper hydroxide WP, was appraised, and the lowest effective concentration was 500 and 75 mg/L, respectively [17]. The control effect of several kinds of copper hydroxide formulation was lower than 50% in cassava plantations located in Guangxi and Hainan provinces (unpublished).

Usually, continuous and widespread application has favored the spread of copper-resistance genes among pathogenic bacterial strains [18,19]. *CopAB*, c*opLAB*, ORF1-4 and other homologous genes are a copper-resistance gene cluster in Xanthomonads [20,21,22,23]. Ryan et al. (2007) isolated a novel gene cluster: *xmeRSA* from *Xanthomonas campestris* strain IG-8. Further research indicates that *xmeRSA* is a homologue of the multidrug efflux system of *Stenotrophomonas maltophilia* and required for a copper-resistant phenotype [24]. Whether Cu^2+^ resistance has likewise begun to emerge in the *X. phaseoli* pv. *manihotis* population and which copper resistance genes plays the important role is worth investigating.

Planting resistant varieties and spraying copper-based bactericides are common methods to control bacterial diseases, but the effect is restricted to CBB. Therefore, the present study aimed to assess the genetic diversity and copper ion resistance of *X. phaseoli* pv. *manihotis*, sequence the homologous genes of copper-resistance gene clusters, and provide a basis for the cultivation of CBB-resistant cassava varieties and further control research on CBB.

## 2. Materials and Methods

### 2.1. Bacterial Strains, Growth Conditions, and Reagents

Forty-eight *X. phaseoli* pv. *manihotis* strains were isolated from diseased leaf or stem samples of China, Uganda, Cambodia, Malaysia, and Micronesia. Following Koch’s postulates, pathogenicity was established by Lu et al. (2013) [25]. *X. phaseoli* pv. *manihotis* strains GLBY06 and GLBY07 were obtained from Universidad de los Andes and all the strains were conserved at the Environment and Plant Protection Institute (EPPI), CATAS. The details of 50 *X. phaseoli* pv. *manihotis* strains are shown in Table 1. These strains were grown on YPGA medium (yeast powder, 5 g; peptone, 5 g; D-glucose, 10 g; deionized water, 1000 mL; agar, 15 g; pH 7.20). Total genomic DNA was prepared by using the procedure of Fargier et al. (2018) [26]. Taq DNA polymerase, PCR buffer (contain MgCl_2_) and dNTPs were purchased from TIANGEN Co., Ltd. (Beijing, China), Primer synthesis and amplicon sequencing were performed by BGI Co., Ltd. (Shenzhen, China). Anhydrous copper (II) sulfate (CuSO_4_) was purchased from Sangon Biotech Co., Ltd. (Shanghai, China). A solution of Cu^2+^ (0.5 M) was created by dissolving anhydrous copper (II) sulfate in ddH_2_O, filtering through a 0.22 μm filter and storing at 4 °C.

### 2.2. Clustering Based on Multilocus Sequence Analysis

Using the methodology described by Bella et al. (2019) [27], genomic DNA of fifty *X. phaseoli* pv. *manihotis* strains was extracted. The PCR reaction contained a mixture of 1 × PCR buffer, 0.1 mM dNTPs, 0.2 μM each of forward and reverse primers, 50–200 ng genomic DNA, 2.0–3.0 U of Taq DNA polymerase, with sterilized distilled water added to 50 μL. All the PCR amplicon was obtained with the primers listed in Table 2. Using the same primers of *efp*, both ends of the amplicon were sequenced and merged, then a partial sequence of *efp* was obtained. Using the primers P-X-atpDF/P-X-atpDR/ATPD-S1/ATPD-S2, P-X-dnaKF/P-X-dnaKR/dnaK-S1/dnaK-S2, X-gyrB1F/X-gyrB1R/gyrB-S1/gyrB-S2, X-gyrB1F/X-gyrB1R/gyrB-S1/gyrB-S2 and X-rpoD1F/X-rpoD1R/rpoD-S1/rpoD-S2 listed in Table 2, the partial sequences of four housekeeping genes—*atpD*, *dnaK*, *gyrB* and *rpoD*—were also obtained. The same five gene sequences from *Xanthomonas axonopodis* pv. *citrumelo* strain F1 (CP002914.1) [28] acted as an outgroup. The phylogenetic tree of *X. phaseoli* pv. *manihotis* strains was constructed using the neighbor-joining method (bootstrap = 1000) using MEGAX software (version 11.0.11) [29].

### 2.3. Copper Tolerance Assays

After autoclaving, YPGA medium was kept in a water bath at 50 °C, and different quantities of CuSO_4_ solution (0.5 M) were thoroughly mixed with the YPGA medium before pouring the medium into plates. To estimate and narrow down the MIC range of Cu^2+^ in *X. phaseoli* pv. *manihotis* strains, five representative strains from different countries—GLBY06, CGX11, CGD24, UGD1, and KHM04—were used in the first round, and the tested Cu^2+^ concentrations were 0, 0.4, 0.8, 1.2, 1.6, 2.0, and 2.4 mM. In the second round, the tested concentrations ranged from 1.2 to 2.0 mM with 0.1 mM increments. All strains were cultured on YPGA medium for 48 h, suspended in sterile water at OD_600_ = 0.5 and streaked on YPGA medium containing CuSO_4_; medium without copper served as the control. The growth of strains was assessed after 72 h of incubation at 28 °C. The experiments were repeated three times. The minimum Cu^2+^ concentration at which a strain failed to grow was determined to be the MIC. On the basis of the results of Cu^2+^ resistance detection on YPGA, six representative strains—CHN03, UGD1, MLXY1825, MK1825, KHM01, and GLBY06—were selected. A 0.1 mL (OD_600_ = 0.5) suspension was prepared and added to 49.9 mL of YPG broth containing different amounts of CuSO_4_, incubated (at 28 °C, 180 rpm), and the OD_600_ value was measured at 48 h, 60 h, and 72 h to reconfirm the MIC.

### 2.4. Analysis of Copper-Resistance-Related Genes

Based on *X. phaseoli* pv. *manihotis* strain CHN01 (CP083575.1) genome annotations [4], the encoded amino acid sequences of *copAB* (AE008923), *copLAB* (AY536748 and HM362782), ORF1-4 (L19222.1) and *xmeRSA* (AY359472.5) were obtained from the GenBank database, and two homologous gene clusters of *copLAB* and *xmeRSA* were anchored. The structure and encoded functional proteins of *copLAB* and *xmeRSA* were analyzed, and other copper-resistance-related genes were also screened.

The primers X-LABF/X-LABR and X-RSAF/X-RSAR (Table 2) were designed to amplify the sequences of *copLAB* and *xmeRSA* from *X. phaseoli* pv. *manihotis* strains in this study. Using the primers X-LABF/X-LABR/copLAB-S1/copLAB-S2/copLAB-S3/copLAB-S4/copLAB-S5, and X-RSAF/X-RSAR/xmeRSA-S1/xmeRSA-S2/xmeRSA-S3/xmeRSA-S4/xmeRSA-S5, listed in Table 2, the amplicon sequences of *copLAB* and *xmeRSA* were obtained.

## 3. Results

### 3.1. Clustering Based on Multilocus Sequence Analysis

**Figure 1:**
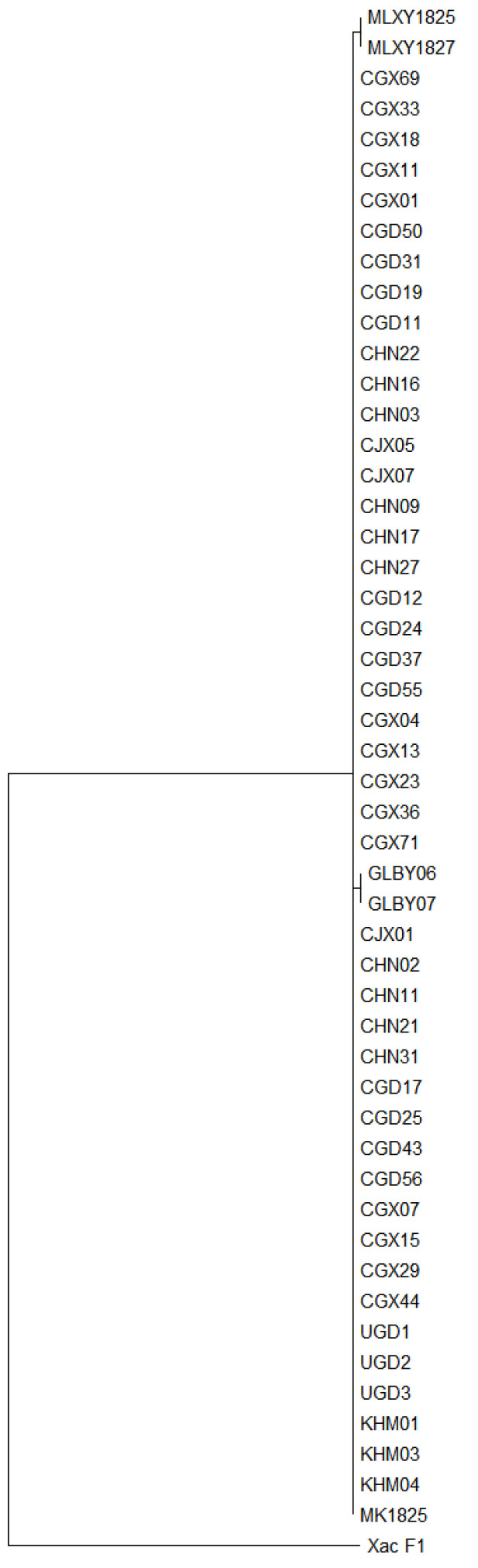
Clustering of 50 *Xanthomonas phaseoli* pv. *manihotis* strains on the basis of multilocus sequence analysis (*atpD*-*dnaK*-*gyrB*-*efp*-*rpoD* concatenated). The phylogenetic tree was constructed using the neighbor-joining method (bootstrap = 1000) using MEGA software (version 11.0.11). Xac F1 (National Center for Biotechnology Information Reference Sequence: CP002914.1), one of the *Xanthomonas axonopodis* pv. *citrumelo* type strains, was used as the outgroup.

The five housekeeping genes *atpD*, *dnaK*, *gyrB*, *efp*, and *rpoD* were successfully amplified from 50 *X. phaseoli* pv. *manihotis* strains (Table 1), with a partial sequence length of 868, 1034, 904, 445 and 959 nucleotides, respectively (Table 2). These five sequences were concatenated in the order *atpD*–*dnaK*–*gyrB*–*efp*–*rpoD*, and the total length was 4210 nucleotides (Supplementary File S1). In the neighbor-joining phylogenetic tree, the 50 strains were clustered into three groups (Figure 1). Group I contained 46 strains from China, Uganda, Cambodia, and Micronesia. Group II contained two strains from Colombia, and Group III contained the remaining two strains from Malaysia.

The five sequenced genes were highly conserved among strains from different geographic regions. *DnaK*, *gyrB* and *efp* had the same sequence in all strains, whereas a few polymorphisms were observed in *atpD* and *rpoD*. One point mutation occurred at position 361 of the trimmed *atpD* sequence: A in Groups I and II and C in Group III. Similarly, for *rpoD*, there was only one nucleotide polymorphism observed between the Group II strains and strains in the other two groups; this mutation was located at position 609 with an A-to-T transversion in the two Group II strains.

### 3.2. Copper Resistance

In the first round, five representative strains were tested to narrow down the Cu^2+^ resistance threshold. The strains grew on solid medium plates or broth culture containing >1.2 mM of Cu^2+^. Only one strain, GLBY06, could grow with 1.6 mM of Cu^2+^. On solid medium, the thickness and size of the colony reduced with increasing Cu^2+^ concentration.

After the second round, the minimal inhibitory concentration (MIC) was determined as the lowest Cu^2+^ concentration at which no growth was observed in plate culture. Six strains—CHN03, UGD1, MLXY1825, MK1825, KHM01, and GLBY06—from different groups and areas were also tested in YPG broth medium, and similar observations were made. The MIC of the 50 *X. phaseoli* pv. *manihotis* strains ranged from 1.3 to 1.7 mM (Table 1). Strains CHN22, CGD31, CGX04, and GLBY06 had the highest MIC value of 1.7 mM, whereas CJX05, CHN02, CGX01, and MLXY1827 had the lowest MIC value of 1.3 mM. The copper-resistance levels of the *X. phaseoli* pv. *manihotis* strains did not differ significantly according to geographic region.

In total, 39 *X. phaseoli* pv. *manihotis* strains had been isolated from diseased samples collected in China. The MIC of copper was 1.3 mM for three strains (CJX05, CHN02 and CGX01), 1.4 mM for 10 strains (CJX01, CJX07, CHN11, CHN21, CGD24, CGD55, CGX18, CGX23, CGX29 and CGX71), 1.5 mM for 12 strains (CHN09, CHN16, CHN17, CGD11, CGD17, CGD43, CGD56, CGX07, CGX15, CGX33, CGX36 and CGX69), 1.6 mM for 11 strains (CHN03, CHN27, CHN31, CGD12, CGD19, CGD25, CGD37, CGD50, CGX11, CGX13 and CGX44), and 1.7 mM for the other three strains (CHN22, CGD31, CGX04), with an average MIC of 1.5 mM.

### 3.3. Copper-Resistance-Related Genes

The homologous gene clusters of *copLAB* and *xmeRSA* of *X. phaseoli* pv. *manihotis* strain CHN01 were identified at two different loci, and no other identifiable copper-resistance-related gene was detected at these two loci. Thirteen other copper-related genes were also screened, including those encoding copper-binding protein, CopD family protein, copper homeostasis protein CutC, and copper-type cytochrome.

**Figure 2:**
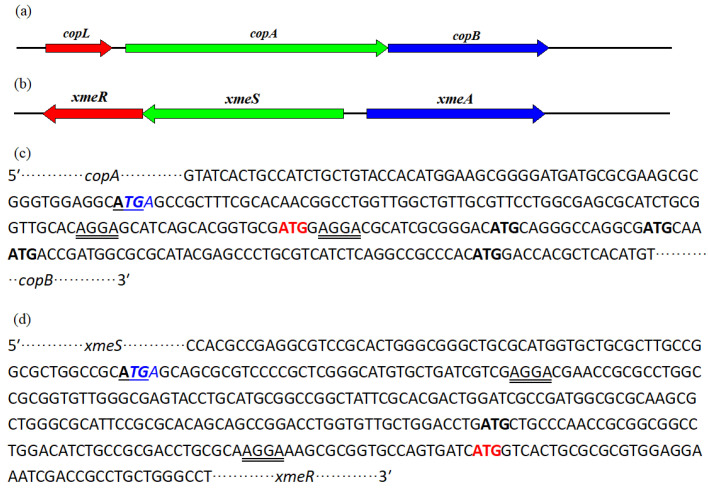
Organization of the gene clusters (**a**) *copLAB* and (**b**) *xmeRSA* and intergenic region (**c**) between *copA* and *copB* and (**d**) between *xmeS* and *xmeR*. The *copA*/*xmeS* stop codon is shown in blue and italics, and the *copB*/*xmeR* start codon is shown in bold and underlined letters. Putative ATG start codons for *copB*/*xmeA* are shown in bold, and the start codon of *copB*/*xmeR* suggested in this study is shown in red. Putative ribosome-binding sites are shown in double-underlined letters.

The full-length *copLAB* gene cluster was 3459 nucleotides, comprising three genes, *copL*, *copA*, and *copB*, each with the same coding direction (Figure 2a). According to the genome annotation, the distance between the stop codon of *copL* and the start codon of *copA* was 88 nucleotides, and the stop codon of *copA* overlapped with the start codon of *copB*. No putative ribosome-binding site (core sequence AGGA; www.ics.uci.edu/~kibler/pubs/TR03 accessed on 2 June 2023) was found at the end of *copL* and *copA* or at the adjacent sequence upstream of the coding region of *copL*. Two putative ribosome-binding sites were identified at the beginning of the *copB* coding region (Figure 2c). The first putative site was located upstream of alternative ATG start codons; however, it was located at a distance of 84 nucleotides from annotated start codons, which suggests that another putative start codon should be considered. *copL* encodes a predicted 152-amino acid metal-binding regulatory protein, *copA* encodes a predicted 601-amino acid multicopper oxidase, and *copB* encodes a predicted 369-amino acid copper resistance protein. The deduced amino acid sequences of *copL*, *copA*, and *copB* exhibited 100% (124/124) identity with that of *copL* from *Xanthomonas axonopodis* pv. *vesicatoria*, 96% (572/594) identity with that of *copA* and 86% (317/369) identity with that of *copB* from *X. axonopodis* pv. *citri* [20,21].

The full-length sequence of the *xmeRSA* gene cluster was 3444 nucleotides, comprising three genes *xmeR*, *xmeS*, and *xmeA*, and the coding direction of *xmeA* was inverse to that of the other two genes (Figure 2b). The annotation results indicated that the distance between the two start codons of *xmeS* and *xmeA* was 160 nucleotides, and the stop codon of *xmeS* overlapped with the start codon of *xmeR*. No putative ribosome-binding site was identified in the adjacent upstream sequence of the coding region of *xmeS* and *xmeA* or at the end of *xmeS*. Two putative ribosome-binding sites were identified at the beginning of the *xmeR* coding region, with the second site located upstream of alternative ATG start codons. The distance from the alternative ATG start codons to the annotated start codons was 243 nucleotides (Figure 2d), suggesting that another putative start codon should be considered. *xmeR* encodes a predicted 227-amino acid transcriptional regulatory protein that exhibits 100% (227/227) identity with the DNA-binding transcriptional regulator BaeR of *Xanthomonas citri* (WP_017157623.1) [32]. *XmeS* encodes a predicted 459-amino acid protein that exhibits 100% (459/459) identity with sensor histidine kinase efflux regulator BaeS of *Xanthomonas phaseoli* (WP_017157622.1). *XmeA* encodes a predicted 407-amino acid protein that exhibits 100% (407/407) identity with the multidrug efflux RND transporter periplasmic adaptor subunit AcrA of *Xanthomonas phaseoli* (WP_017157621.1). The predicted amino acid sequences of *xmeR*, *xmeS*, and *xmeA* also showed 67% (151/227), 57% (260/455), and 61% (115/189) identity with *xmeR*, *xmeS*, and *xmeA* of *Xanthomonas campestris* strain IG-8 (AY359472.5), respectively, which are also associated with the copper resistance [24].

### 3.4. Two Gene Clusters Were Highly Conserved

*CopLAB* and *xmeRSA* play an important role in the copper-resistant *Xanthomonads* strains, so these two gene clusters were chosen as the target for further analysis. *copLAB* and *xmeRSA* were also successfully amplified from 50 *X. phaseoli* pv. *manihotis* strains. Polymorphisms in the two gene clusters comprised the few differences among the determined sequences. For *copLAB*, only one point substitution at position 963 with a G-to-A transversion in the coding region of *copA* but no amino acid transversion, was observed in strain CGD17. The other 49 strains had the same *copLAB* sequence as strain CHN01 (Supplementary File S2).

The sequence of *xmeRSA* in the seven strains CHN02, CHN09, CHN17, CHN21, CGD37, CGX04, and CGX69 exhibited 100% identity with that in strain CHN01. Two point substitutions were observed in strain CGD50: a C-to-A transversion at position 1160 in the coding region of *xmeA*, leading to an amino acid change from glycine to valine and an A to G transversion at position 1015 in the coding region of *xmeS*, leading to amino acid change from threonine to alanine. The remaining 42 strains showed the same single point substitution: an A-to-G transversion at position 1015 in the coding region of *xmeS* (Supplementary File S3).

## 4. Discussion

CBB frequently causes severe losses in cassava plantations in China and has become the most damaging disease of cassava. Remarkably, copper-based bactericides have failed to control this disease, and the main cassava cultivars are susceptible. The genetic diversity and emergent copper-ion resistance of *X. phaseoli* pv. *manihotis* are major obstacles to the field management of CBB in China. As such, the genetic diversity and copper resistance of *X. phaseoli* pv. *manihotis* population was studied. Fifty strains were used in this study, thirty-nine strains originating from four provinces of China respectively, and eleven strains from five other countries.

The genetic diversity of 50 *X. phaseoli* pv. *manihotis* strains from different geographic regions was assessed by sequencing the five housekeeping genes *atpD*, *dnaK*, *gyrB*, *efp*, and *rpoD*. All the strains showed low genetic diversity and a certain degree of correlation with the geographical origin. This is similar to the genetic diversity of *X. phaseoli* pv. *manihotis* strains assessed on the basis of concatenated SNPs [13]. In the present study, the strains from the Asian mainland (China and Cambodia), Africa (Uganda), and Central Pacific Region (Micronesia) clustered into the same group. Unlike Uganda or Micronesia, Malaysia is located in the Asian continent, but the only two strains from Malaysia clustered into a separate group (Group III).

The partial sequences of *dnaK*, *gyrB*, and *efp* genes were highly conserved, and no nucleotide substitutions were found among these strains. In fact, a phylogenetic analysis using the concatenated sequence of the two genes *atpD* and *rpoD* would produce the same clustering groups. The *gyrB* sequences were highly conserved, with the same sequence from 60 *X. arboricola* pv. *juglandis* isolates from China [33]. The genetic diversity analysis of 59 *X. arboricola* pv. *juglandis* isolates from Serbia also showed that this gene was not a good choice to assess genetic diversity. Our results showed only one nucleotide polymorphism observed among different group strains, implying that all these five genes were unsuitable for population structure analysis of *X. phaseoli* pv. *manihotis*.

The pressure imposed by the continuous application of copper-based bactericides drives the selection of copper-resistant strains and favors a gradual increase in the frequency of resistant pathogens within the bacterial population. Garde et al. (1991) collected and studied many *Xanthomonas campestris* pv. *vesicatoria* strains from Florida, New Mexico, Oklahoma and California, USA, and found that copper-resistant strains had emerged in the environment and the number had increased owing to the heavy use of copper-based bactericidal sprays [34]. In Australia, copper-based bactericides are the only products available to pepper producers to combat bacterial spot, and the number and proportion of copper-resistant *X. campestris* pv. *vesicatoria* strains have increased with the increasing application frequency. In Queensland, only one *X. campestris* pv. vesicatoria strain (of 12) collected before 1987 could tolerate 1.0 mM Cu^2+^. In contrast, approximately one in four strains (75 total) collected between 1999 and 2000 tolerated more than 1.0 mM Cu^2+^, and the MIC for all four strains isolated in 2015 was 1.5 or 2.0 mM Cu^2+^ [35,36]. In Hubei, China, where walnut bacterial blight caused by *X. arboricola* pv. *juglandis* was common, the high ratio of copper-resistant isolates was also associated with the heavy use of copper-based bactericides. Two isolates from Danjiangkou, where copper compounds had been frequently applied for more than 10 years, were extremely highly resistant to Cu^2+^ (270 µg/mL) [33].

Copper-resistant *Xanthomonas* strains have been found worldwide. In a previous study, 87 of 98 *Xanthomonas* strains causing tomato bacterial spot collected in Ontario, Canada were reported to grow on mannitol–glutamate–yeast extract (MGY) medium containing 1.0 mmol per liter of Cu^2+^ or higher [37]. Using a Cu^2+^ concentration of 125 µg/mL (0.5 mM) as the threshold dose, Fu et al. (2021) categorized *X. arboricola* pv. *juglandis* isolates into resistant and sensitive groups [33]. Previous studies have reported that only some of the studied strains of plant pathogenic bacteria were copper-resistant. However, in the present study, the MIC of all the fifty *X. phaseoli* pv. *manihotis* strains was 1.3 to 1.7 mM, indicating moderate levels of copper resistance. These findings explain why copper-based bactericides were ineffective in CBB control. Notably, there was no evident relationship between the level of copper resistance and genetic diversity based on MLSA.

In China, copper compounds were only occasionally used to control CBB in the field. In this paper, we found all the strains could tolerate 1.3 mM Cu^2+^. To our knowledge, there are almost no records of the use of copper-based bactericides in Uganda, Cambodia, and other countries included in this study. Thus, it remains unknown why all the *X. phaseoli* pv. *manihotis* strains exhibit resistance to Cu^2+^.

Via conjugation, plasmid-borne copper-resistance gene clusters may be horizontally transferred to different *Xanthomonas* strains [19]. *CopLAB*, a typical copper-resistance gene cluster, is often encoded by plasmid and is present in many copper-resistant *Xanthomonas* species isolated from different parts of the world. *Xanthomonas* strain IG-8 isolated from soil collected in Hebei province (China) was heavily contaminated with glyphosates and several kinds of heavy metals. *XmeRSA* genes play a major role in the copper-, arsenic-, and cadmium-resistant phenotype, and bioinformatics analysis suggests that these genes were acquired by horizontal gene transfer [24]. The genome annotation of *X. phaseoli* pv. *manihotis* strain CHN01 confirmed that *copLAB* and *xmeRSA* were encoded in the chromosome. The predicted amino acid sequences of these two gene clusters from 50 *X. phaseoli* pv. *manihotis* strains showed high homology, indicating that these two gene clusters were also acquired from other *Xanthomonas* strains. According to the genome annotation of *X. axonopodis* pv. *citri* strain 306, the stop codon of *copA* overlaps with the start codon of *copB* [20]. The same overlaps happened on the *copA* and *xmeS* from *X. phaseoli* pv. *manihotis* strains in this paper, while the coding direction of *xmeS* was inverse to the homologous gene of *Xanthomonas* strain IG-8 [24].

*CopA* and *copB* encode multicopper oxidase and copper-resistance proteisn, whereas *copL* encodes a copper-binding protein to regulate the expression of *copA* and *copB*. The *xmeRSA* from *X. phaseoli* pv. *manihotis* strains is homologous to *xmeRSA* from *Xanthomonas* strain IG-8, which encodes a multidrug efflux system and plays a key role in the multiresistant phenotype. In the present study, all 50 *X. phaseoli* pv. *manihotis* strains showed moderate levels of copper resistance and carried two different copper-resistance-related gene clusters, suggesting that the regulation of copper resistance is associated with two distinct metabolic pathways.

Some copper-resistance genes are also required for full virulence of *Xanthomonas* strains. A *copA* mutation of *X. campestris* pv. *campestris* strain Xc17 resulted in a significant decrease in copper tolerance and virulence on cabbage [38]. *Xanthomonas gardneri* strain Xv10 isolated from diseased tomato leaves was collected in the Inner Mongolia province of China, and the virulence on tomato and level of copper resistance declined with *copB* deletion [39]. The *X. phaseoli* pv. *manihotis* strains used in the present study were resistant to Cu^2+^ without high selective pressure in the field, suggesting that *copLAB* and *xmeRSA* also have another conserved function. The functions of *copLAB* and *xmeRSA* need to be further investigated in future studies.

## 5. Conclusions

Fifty *X. phaseoli* pv. *manihotis* strains were collected from China, Uganda, Cambodia, Colombia, Malaysia, and Micronesia. Using multilocus sequence analysis of five housekeeping genes (*atpD*–*dnaK*–*gyrB*–*efp*–*rpoD*), the strains clustered into three groups. The largest was group I, comprising 46 strains from China, Uganda, Cambodia, and Micronesia, while the other two groups comprised four strains from Colombia and Malaysia separately. Without the pressure imposed by the continuous application of copper-based bactericides, all the strains were resistant to copper, and the copper-resistance levels of the *X. phaseoli* pv. *manihotis* strains did not differ significantly according to geographic region. Copper-based bactericides are not a good choice for CBB, while ethylicin and 2-amino-5-sulfydryl-1,3,4-thiadiazole zinc were effective [40]. Two conservative copper-resistance-related gene clusters, *copLAB* and *xmeRSA*, were amplified from all strains, suggesting they also have another conserved function. In addition, the structural composition of two gene clusters was analyzed.

## Figures and Tables

**Table 1:** *Xanthomonas phaseoli* pv. *manihotis* strains used in this study.

NO.	Strain	Year of Samples Collected	Source	MIC ^a^ (mM)
1	CJX01	2013	Dongxiang, Jiangxi, China	1.4
2	CJX05	2015	Dongxiang, Jiangxi, China	1.3
3	CJX07	2020	Fuzhou, Jiangxi, China	1.4
4	CHN02	2009	Danzhou, Hainan, China	1.3
5	CHN03	2009	Qionghai, Hainan, China	1.6
6	CHN09	2009	Wenchang, Hainan, China	1.5
7	CHN11	2015	Qiongzhong, Hainan, China	1.4
8	CHN16	2017	Haikou, Hainan, China	1.5
9	CHN17	2017	Danzhou, Hainan, China	1.5
10	CHN21	2017	Chengmai, Hainan, China	1.4
11	CHN22	2017	Wenchang, Hainan, China	1.7
12	CHN27	2018	Danzhou, Hainan, China	1.6
13	CHN31	2019	Chengmai, Hainan, China	1.6
14	CGD11	2008	Suixi, Guangdong, China	1.5
15	CGD12	2009	Yangjiang, Guangdong, China	1.6
16	CGD17	2009	Kaiping, Guangdong, China	1.5
17	CGD19	2012	Zhanjiang, Guangdong, China	1.6
18	CGD24	2013	Yunfu, Guangdong, China	1.4
19	CGD25	2015	Guangzhou, Guangdong, China	1.6
20	CGD31	2016	Leizhou, Guangdong, China	1.7
21	CGD37	2016	Suixi, Guangdong, China	1.6
22	CGD43	2017	Yunfu, Guangdong, China	1.5
23	CGD50	2017	Jiangmen, Guangdong, China	1.6
24	CGD55	2019	Kaiping, Guangdong, China	1.4
25	CGD56	2020	Leizhou, Guangdong, China	1.5
26	CGX01	2008	Wuming, Guangxi, China	1.3
27	CGX04	2008	Hepu, Guangxi, China	1.7
28	CGX07	2009	Dongxing, Guangxi, China	1.5
29	CGX11	2010	Beihai, Guangxi, China	1.6
30	CGX13	2015	Pingnan, Guangxi, China	1.6
31	CGX15	2015	Guiping, Guangxi, China	1.5
32	CGX18	2016	Nanning, Guangxi, China	1.4
33	CGX23	2016	Beihai, Guangxi, China	1.4
34	CGX29	2017	Wuxuan, Guangxi, China	1.4
35	CGX33	2017	Hepu, Guangxi, China	1.5
36	CGX36	2018	Nanning, Guangxi, China	1.5
37	CGX44	2019	Longan, Guangxi, China	1.6
38	CGX69	2020	Pingnan, Guangxi, China	1.5
39	CGX71	2020	Guiping, Guangxi, China	1.4
40	UGD1	2014	Kiryandongo, Uganda	1.4
41	UGD2	2014	Masindi, Uganda	1.5
42	UGD3	2015	Hoima, Uganda	1.6
43	KHM01	2016	Kratié, Cambodia	1.4
44	KHM03	2017	Kratié, Cambodia	1.6
45	KHM04	2018	Stung Treng, Cambodia	1.4
46	MLXY1825	2018	Sabah, Malaysia	1.4
47	MLXY1827	2018	Sabah, Malaysia	1.3
48	MK1825	2018	Micronesia	1.5
49	GLBY06	2013	Colombia	1.7
50	GLBY07	2013	Colombia	1.4

^a^ MIC, minimal inhibitory concentration.

**Table 2:** Primers used in this study.

Gene/Gene Cluster	Coding Protein	Primer Name	Sequence (5′–3′) ^b^	Size of Amplicon	Reference
*atpD*	ATP synthase β-chain	P-X-atpDF	GGGCAAGATCGTTCAGAT	868 nt	Fischer-Le Saux et al. (2015) [30].
		P-X-atpDR	GCTCTTGGTCGAGGTGAT		
		ATPD-S1	**CTGGAAACCGGCATCAAGGTC**		This study
		ATPD-S2	**CGTGGTAGAAGTCGTTGCCCT**		
*dnaK*	Molecular chaperone DnaK (HSP70)	P-X-dnaKF	GGTATTGACCTCGGCACCAC	1034 nt	Fischer-Le et al. (2015) [30].
		P-X-dnaKR	ACCTTCGGCAT**A**CGGGTCT		
		dnaK-S1	**ACTTCAACGACAGCCAG**		This study
		dnaK-S2	**TACTCGATGACGCGGTTGT**		
*gyrB*	DNA topoisomerase β-subunit	X-gyrB1F	ACGAGTACAACCCGGACAA	904 nt	Fischer-Le et al. (2015) [30].
		X-gyrB1R	CCCATCA**A**GGTGCTGAAGAT		
		gyrB-S1	**TTGTTGATGCTGTTCACCA**		This study
		gyrB-S2	**CTCTTGTTGCCGCGCACGATCT**		
*efp*	elongation factor P	X-efp1F	TCATCACCGAGACCGAAT	445 nt	Hamza et al. (2012) [31].
		X-efp1R	TCCTGGTTGACGAACAG		
*rpoD*	RNA polymerase sigma factor	X-rpoD1F	**TGGTCAACGGCATCAAGGA**	959 nt	This study
		X-rpoD1R	**CAACGCCTTGGCCTCTATCT**		
		rpoD-S1	**TCAAGGACCAGATCATCTCC**		
		rpoD-S2	**TCGATCATGTGCACCGGGATAC**		
*copLAB*	metal-binding regulatory protein, multicopper oxidase and copper resistance protein	X-LABF X-LABR	**ATGCTATGCTCGCTGCACCT**	3459 nt	This study
			**TCAAAACCACACGCGTACAC**		
		copLAB-S1	**AGGAGATGTCATGTCGTTCGAT**		
		copLAB-S2	**AGCCACGACAACTACGCACA**		
		copLAB-S3	**GCAACCCGCTGATCGACAT**		
		copLAB-S4	**TACGCAAGCACACCATCGACAT**		
		copLAB-S5	**AACGACACGACGGACGCACCGAAT**		
*xmeRSA*	transcriptional regulatory protein, sensor histidine kinase efflux regulator and multidrug efflux RND transporter periplasmic adaptor subunit	X-RSAF X-RSAR	**ATTCCTCAGTCGGCCTTGG**	3444 nt	This study
			**TCACGGCTCAAACCGATACC**		
		xmeRSA-S1	**CAGAGCGAGGTGCTGGCCAC**		
		xmeRSA-S2	**TGTAGTCGAGATTGATGCGC**		
		xmeRSA-S3	**TTACCGCGCTTCGCATCACCTC**		
		xmeRSA-S4	**CAAGCGCTGGAGCACAACGAA**		
		xmeRSA-S5	**ATGTGCTGATCGTCGAGGA**		

^b^ The nucleotides and sequences in bold were revised or designed according to the published sequences of *X. phaseoli* pv. *manihotis* strain (CP083575.1) from the GenBank database.

## Data Availability

Not applicable.

## Supplementary Materials

The following supporting information can be downloaded at https://www.mdpi.com/article/10.3390/cimb45070342/s1. Supplementary File S1: Partial sequence of *atpD*-*dnaK*-*gyrB*-*efp*-*rpoD*; Supplementary File S2: Sequence of *copLAB*; Supplementary File S3: Sequence of *xmeRSA.*

## References

[1] Faostat (2021). FAOSTAT Statistical Database. http://www.fao.org/faostat/en/#data/QCL.

[2] Commonwealth Agricultural Bureaux International(CABI) Digital Library *Xanthomonas axonopodis* pv. *manihotis* (Cassava Bacterial Blight). https://www.cabidigitallibrary.org/doi/10.1079/cabicompendium.56952.

[3] Mansfield J., Genin S., Magori S., Citovsky V., Sriariyanum M., Ronald P., Dow MA X., Verdier V., Beer S.V., Machado M.A. (2012). Top 10 plant pathogenic bacteria in molecular plant pathology. Mol. Plant Pathol..

[4] Zhu S.-S., Pan Y.-Y., Li K., Fan R.-C., Xiang L., Huang S.-Y., Jia S.-H., Niu X.-L., Li C.-X., Chen Y.-H. (2022). Complete genome sequence of *Xanthomonas phaseoli* pv. *manihotis* strain CHN01, the causal agent of cassava bacterial blight. Plant Dis..

[5] Wen Y.-T. (1982). Pathogen identification of cassava bacterial blight. Chin. J. Trop. Crops.

[6] Li C.-P., Shi T., Liu X.-B., Cai J.-M., Pei Y.-L., Huang G.-X. (2011). General survey on cassava diseases and safety assessment of cassava bacterial blight. Chin. J. Trop. Crops.

[7] Da Gama M.A.S., Mariano R.D.L.R., da Silva J.W.J., de Farias A.R.G., Barbosa M.A.G., da Silva Velloso Ferreira M.A., Costa C.R.L., Santos L.A., de Souza E.B. (2018). Taxonomic repositioning of *Xanthomonas campestris* pv. *viticola* (Nayudu 1972) Dye 1978 as *Xanthomonas citri* pv. *viticola* (Nayudu 1972) Dye 1978 Comb. nov. and emendation of the description of *Xanthomonas citri* pv. *anacardii* to include pigmented isolates pathogenic to cashew plant. Phytopathology.

[8] Stackebrandt E., Frederiksen W., Garrity G.M., Grimont P.A.D., Kampfer P., Maiden M.C.J., Nesme X., Rossello-Mora R., Swings J., Truper H.G. (2002). Report of the ad hoc committee for the re-evaluation of the species definition in bacteriology. Int. J. Syst. Evol. Microbiol..

[9] Louws F.J., Rademaker J.L.W., de Bruijn F.J. (1999). The three DS of PCR-based genomic analysis of phytobacteria: Diversity, detection, and disease diagnosis. Annu. Rev. Phytopathol..

[10] Ntambo M.S., Meng J.-Y., Rott C., Royer M., Lin L.-H., Zhang H.-L., Gao S.-J. (2019). Identification and characterization of *Xanthomonas albilineans* causing sugarcane leaf scald in China using multilocus sequence analysis. Plant Pathol..

[11] Chen G., Kong C.-C., Yang L.-M., Zhuang M., Zhang Y.-Y., Wang Y., Ji J.-L., Fang Z.-Y., Lv H.-H. (2021). Genetic diversity and population structure of the *Xanthomonas campestris* pv. *campestris* strains affecting cabbages in China revealed by MLST and Rep-PCR based genotyping. Plant Pathol. J..

[12] Restrepo S., Vélez C.M., Erdier V.V. (2000). Measuring the genetic diversity of *Xanthomonas axonopodis* pv. *manihotis* within different fields in Colombia. Phytopathology.

[13] Bart R., Cohn M., Kassen A., McCallum E.J., Shybut M., Petriello A., Krasileva K., Dahlbeck D., Medina C., Alicai T. (2012). High-throughput genomic sequencing of cassava bacterial blight strains identifies conserved effectors to target for durable resistance. Proc. Natl. Acad. Sci. USA.

[14] Verdier V., Restrepo S., Mosquera G., Jorge V., López Carrascal C.E. (2004). Recent progress in the characterization of molecular determinants in the *Xanthomonas axonopodis* pv. *manihotis*-cassava interaction. Plant Mol. Biol..

[15] Restrepo S., Vélez C.M., Duque M.C. (2004). Genetic structure and population dynamics of *Xanthomonas axonopodis* pv. *manihotis* in Colombia from 1995 to 1999. Appl. Environ. Microbiol..

[16] Ogunjobi A.A., Fagade O.E., Dixon A.G.O. (2010). Comparative analysis of genetic variation among *Xanthomonas axonopodis* pv *manihotis* isolated from the western states of Nigeria using RAPD and AFLP. Indian J. Microbiol..

[17] Lu X., Li C.-P., Shi T., Huang G.-X. (2013). Bactericide screening against pathogen of cassava bacterial blight. Chin. J. Trop. Agric..

[18] Cooksey D.A., Azad H.R., Cha J.-S., Lim C.-K. (1990). Copper resistance gene homologs in pathogenic and saprophytic bacterial species from tomato. Appl. Environ. Microbiol..

[19] Franklin B., Jason C.H., Jeffrey B.J., James H.G. (2013). Evidence for acquisition of copper resistance genes from different sources in citrus-associated Xanthomonads. Phytopathology.

[20] Teixeira E.C., de Oliveira J.C.F., Novo M.T.M., Bertolini M.C. (2008). The copper resistance operon copAB from *Xanthomonas axonopodis* pathovar citri: Gene inactivation results in copper sensitivity. Microbiology.

[21] Voloudakis A.E., Reignier T.M., Cooksey D.A. (2005). Regulation of resistance to copper in *Xanthomonas axonopodis* pv. *vesicatoria*. Appl. Environ. Microbiol..

[22] Behlau F., Canteros B.I., Minsavage G.V., Jones J.B., Graham J.H. (2011). Molecular characterization of copper resistance genes from *Xanthomonas citri* subsp. *citri* and *Xanthomonas alfalfae* subsp. *citrumelonis*. Appl. Environ. Microbiol..

[23] Lee Y.-A., Hendson M., Panopoulos N.-J., Schroth M.-N. (1994). Molecular cloning, chromosomal mapping, and sequence analysis of copper resistance genes from *Xanthomonas campestris* pv. *juglandis*: Homology with small blue copper proteins and multicopper oxidase. J. Bacteriol..

[24] Ryan R.P., Ryan D.J., Sun Y.-C., Li F.-M., Wang Y.-P., David N.D. (2007). An acquired efflux system is responsible for copper resistance in Xanthomonas strain IG-8 isolated from China. FEMS Microbiol. Lett..

[25] Lu X., Li C.-P., Shi T., Cai J.-M., Huang G.-X. (2013). Pathogen identification of cassava bacterial blight from several main cultivation area in China. Guangdong Agric. Sci..

[26] Fargier E., Fischer-Le Saux M., Manceau C. (2011). A multilocus sequence analysis of *Xanthomonas campestris* reveals a complex structure within crucifer-attacking pathovars of this species. Syst. Appl. Microbiol..

[27] Bella P., Moretti C., Licciardello G., Strano C.P., Pulvirenti A., Alaimo S., Zaccardelli M., Branca F., Buonaurio R., Vicente J.G. (2019). Multilocus sequence typing analysis of Italian *Xanthomonas campestris* pv. *campestris* strains suggests the evolution of local endemic populations of the pathogen and does not correlate with race distribution. Plant Pathol..

[28] Jalan N., Aritua V., Kumar D., Yu F., Jones J.B., Graham J.H., Setubal J.C., Wang N. (2011). Comparative genomic analysis of *Xanthomonas axonopodis* pv. *citrumelo* F1, which causes citrus bacterial spot disease, and related strains provides insights into virulence and host specificity. J. Bacteriol..

[29] Kumar S., Stecher G., Li M., Knyaz C., Tamura K. (2018). MEGA X: Molecular evolutionary genetics analysis across computing platforms. Mol. Biol. Evol..

[30] Fischer-Le S.M., Bonneau S., Essakhi S., Manceau C., Jacques M.A. (2015). Aggressive emerging pathovars of *Xanthomonas arboricola* represent widespread epidemic clones distinct from poorly pathogenic strains, as revealed by multilocus sequence typing. Appl. Environ. Microbiol..

[31] Hamza A.A., Robene-Soustrade I., Jouen E., Lefeuvre P., Chiroleu F., Fisher-Le S.M., Gagnevin L., Pruvost O. (2012). Multilocus sequence analysis and amplified fragment length polymorphism-based characterization of Xanthomonads associated with bacterial spot of tomato and pepper and their relatedness to *Xanthomonas* species. Syst. Appl. Microbiol..

[32] Pao G.M., Saier M.H. (1995). Response regulators of bacterial signal transduction systems: Selective domain shuffling during evolution. J. Mol. Evol..

[33] Fu B., Zhu J., Lee C., Wang L. (2021). Multilocus sequence analysis and copper ion resistance detection of 60 *Xanthomonas arboricola* pv. *juglandis* isolates from China. Plant Dis..

[34] Garde S., Bender C.L. (1991). DNA probes for detection of copper resistance genes in *Xanthomonas campestris* pv. *vesicatoriat*. Appl. Environ. Microbio..

[35] Roach R., Mann R., Gambley G.G., Shivas R.G., Rodoni B. (2018). Identification of *Xanthomonas* species associated with bacterial leaf spot of tomato, capsicum and chilli crops in eastern Australia. Eur. J. Plant Pathol..

[36] Roach R., Mann R., Gambley G.G., Shivas R.G., Chapman T., Rodoni B. (2020). Pathogenicity and copper tolerance in Australian *Xanthomonas* species associated with bacterial leaf spot. Crop Prot..

[37] Pervaiz A.A., Salah E.K., Brian W., Liang Z. (2015). Occurrence of copper-resistant strains and a shift in *Xanthomonas* spp. causing tomato bacterial spot in Ontario. Can. J. Microbiol..

[38] Hsiao Y.M., Liu Y.F., Lee P.Y., Hsu P.C., Tseng S.Y., Pan Y.C. (2011). Functional characterization of *copA* gene encoding multicopper oxidase in *Xanthomonas campestris* pv. *campestris*. J. Agric. Food Chem..

[39] Wu Y.-N. (2012). Cloning and Functional Identification of the Copper Resistance Gene *copB* in *Xanthomonas gardneri*. Ph.D. Thesis.

[40] Xu C.-H., Shi T., Wang G.-F., Li C.-P., Cai J.-M., Huang G.-X. (2019). Evaluation of control effect of four new pesticides against cassava bacterial blight. Chin. J. Trop. Agric..

